# Local Vibration Stimuli Induce Mechanical Stress-Induced Factors and Facilitate Recovery From Immobilization-Induced Oxidative Myofiber Atrophy in Rats

**DOI:** 10.3389/fphys.2019.00759

**Published:** 2019-06-20

**Authors:** Fusako Usuki, Masatake Fujimura, Atsushi Nakamura, Jiro Nakano, Minoru Okita, Itsuro Higuchi

**Affiliations:** ^1^ Department of Clinical Medicine, National Institute for Minamata Disease, Kumamoto, Japan; ^2^ Basic Medical Sciences, National Institute for Minamata Disease, Kumamoto, Japan; ^3^ Department of Physical Therapy Science, Nagasaki University Graduate School of Biomedical Sciences, Nagasaki, Japan; ^4^ Department of Physical Therapy, Faculty of Medicine, School of Health Sciences, Kagoshima University, Kagoshima, Japan

**Keywords:** oxidative myofiber atrophy, local vibration, therapeutics, mechano-growth factor, mechanotransduction, YAP1, satellite cell

## Abstract

Muscle atrophy can be caused by unloading stress such as microgravity environments or cast immobilization. Therapies for such disuse muscle atrophy and their underlying mechanisms are incompletely understood. Here, we investigated the therapeutic effects of local vibration stimulation on immobilization-induced skeletal muscle atrophy. A rat model was made by placing the left hindlimb in a cast for 1 week, leading to oxidative myofiber atrophy without myopathic changes in soleus skeletal muscle. Vibration stimulus (90 Hz, 15 min) to the plantar fascia of the atrophic hindlimb was performed once a day using a hand-held vibration massager after removal of a cast at the end of the immobilization period. After 2 weeks, rats were dissected, and quantitative analysis of the cross-sectional areas of soleus myofibers was performed. The results revealed that vibration induced significant recovery from disuse muscle atrophy, compared with untreated immobilized samples. Furthermore, vibration treatment suppressed the fiber transition from slow to fast fiber types compared with vibration-untreated immobilized samples. Western blotting analyses of mechanical stress-induced factors revealed that the expression of mechano-growth factor (MGF), systemic insulin-like growth factor I, and the mechanotransduction protein, Yes-associated protein 1 (YAP1), was decreased in untreated immobilized soleus muscle, whereas vibration stimulation restored their expression. No change in the level of phosphorylation of YAP1^Ser127^ was observed, leading to no change in p-YAP1/YAP1 ratio in vibration-treated immobilized soleus muscle. The results indicate that vibration stimulus is effective to restore immobilization-induced inactivation of YAP1 pathway. Phosphorylation of ERK 1/2, but not AKT, was enhanced in vibration-treated immobilized soleus muscle. Furthermore, vibration stimuli restored immobilization-induced downregulation of the paired box transcription factor, PAX7, a critical factor for regenerative myogenesis in muscle satellite cells. Our results indicate that cyclic vibration stimuli are effective in activating satellite cells and facilitate recovery from immobilization-induced oxidative myofiber atrophy through upregulation of MGF and YAP1.

## Introduction

Unloading stress, such as that experienced in the microgravity of space flight or during cast immobilization, induces skeletal muscle atrophy. This disuse muscle atrophy is more severe in slow twitch oxidative fibers than type II fast twitch glycolytic fibers (Thomason and Booth, 1990; Allen et al., 1996; Fitts et al., 2001). The mechanism underlying disuse atrophy remains to be clarified, and effective prevention and treatment are not yet sufficiently developed. Limb immobilization can be modeled in rats using a cast (Hamaue et al., 2015) or by hindlimb suspension (Thomason and Booth, 1990), which causes severe atrophy of soleus skeletal muscle, consisting of slow twitch oxidative fibers. Since the majority of soleus skeletal muscle fibers in rats consist of slow twitch oxidative myofibers (Pullen, 1977), these immobilized rat models are useful for investigation of the mechanisms, prevention, and treatment of disuse skeletal muscle atrophy caused by weightlessness.

Skeletal muscle atrophy can occur in both diseased and healthy people, as a result of unloading stresses or normal aging processes. Skeletal muscle fibers are classified as type I and type II, based on labeling of myosin, and type II has the subtypes, IIa, IIb, and IId/x (Schiaffino and Reggiani, 2011; Wang and Pessin, 2013). Various stresses cause different types of muscle fiber atrophy: aging processes mainly cause atrophy of type II fibers, while unloading stress leads to type I fiber atrophy. In addition, stress-induced fiber transition, from slow oxidative to fast glycolytic fiber types, has been reported in unloading models such as the environment of spaceflight/microgravity and hindlimb suspension (Baldwin et al., 2013); however, the mechanisms underlying different fiber type atrophy, fiber transition, and the regenerative processes remain unknown, although some associated factors such as mechano growth factor (MGF), systemic variant of insulin-like growth factor (IGF-I), Hippo pathway, and muscle stem cells have been reported (Goldspink, 1999; Kandalla et al., 2011; Brooks and Myburgh, 2014; Gnimassou et al., 2017; Fukada, 2018).

Here, we investigated the therapeutic effects of local vibration stimulation of the plantar fascia on immobilization-induced skeletal muscle atrophy. It was previously reported that tendon vibration, applied during hindlimb unloading, can attenuate soleus muscle atrophy (Falempin and In-Albon, 1999). Furthermore, stimulation of the sole cutaneous mechanoreceptors can partially prevent the soleus muscle atrophy developed after 14 days in hindlimb unloading condition (De-Doncker et al., 2000). However, no study of the therapeutic effects of local vibration on immobilization-induced atrophied muscle or the mechanical effects of vibration on the biochemical properties of atrophied muscle has been reported. Regarding the effect of vibration stimuli on other immobilization-induced clinical features, preventive effects on immobilization-induced hypersensitivity in rats (Hamaue et al., 2015), or immobilization-induced decrease in bone mineral density of the vertebra, tibia, and femur in rats (Huang et al., 2018), have been reported. Clinically, we found that vibration stimulus of the plantar fascia is effective against plantar pain and spasticity of the lower limbs of patients with fetal-type Minamata disease (Usuki and Tohyama, 2011, 2016). Since local vibration stimulation is a non-invasive method, investigation of its effectiveness for treatment of atrophied muscle and the underlying mechanism is warranted, with the aim of developing a therapeutic approach for disuse atrophy such as a device for patients previously reported (Canu et al., 2016). Our results demonstrate that local vibration stimulation of the plantar fascia facilitates recovery from immobilization-induced oxidative myofiber atrophy. We further demonstrate that the therapeutic effects of vibration stimuli may be attributable to upregulation of the mechanical stress-induced factors, MGF, and Yes-associated protein 1 (YAP1), which can induce expression of the paired box transcription factor (PAX7), an indispensable factor for regenerative myogenesis in muscle satellite cells.

## Materials and Methods

### Animals and Experimental Design

**Figure 1:**
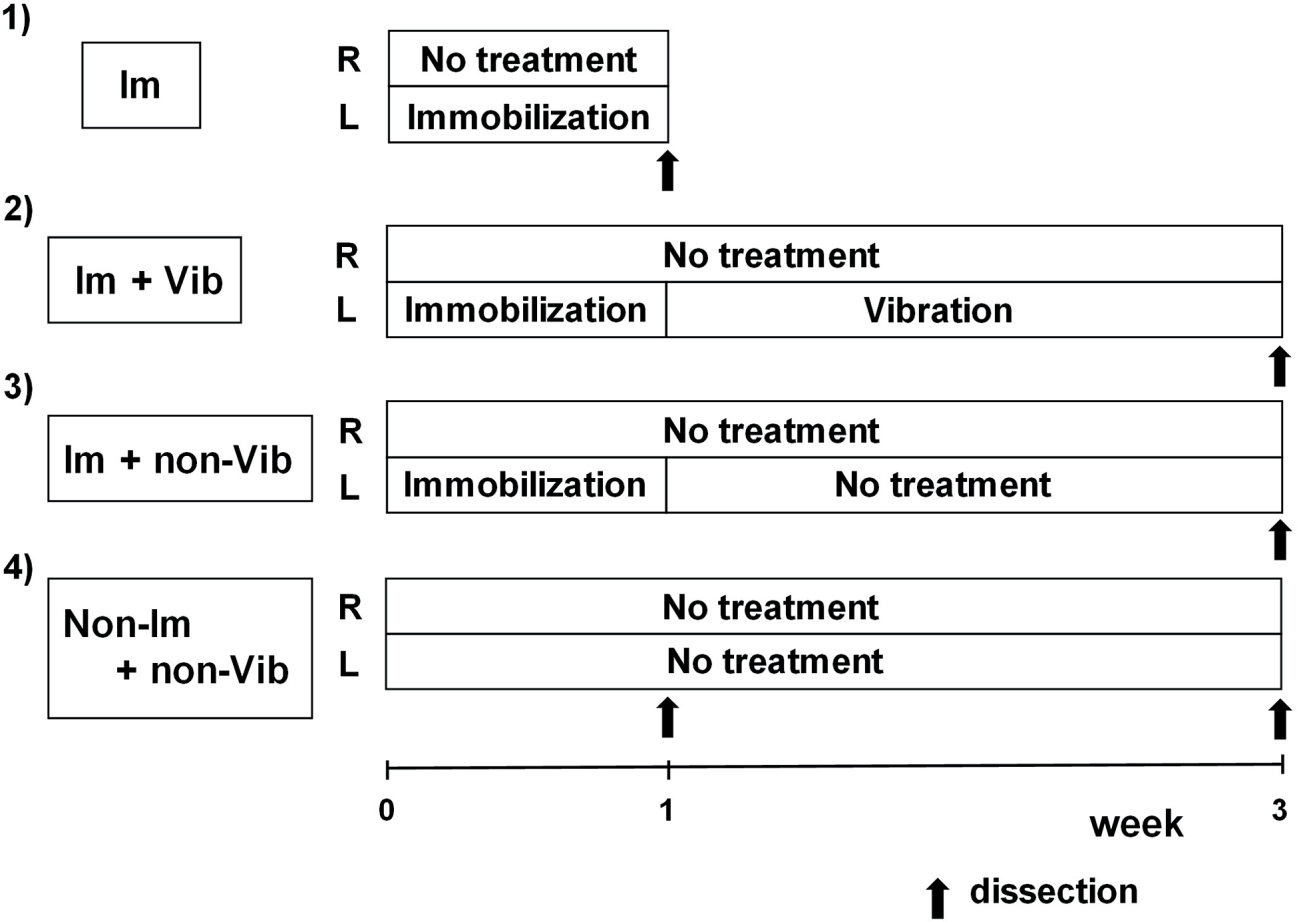
Experimental protocol. Only left limbs were immobilized for 1 week; the contralateral (right) limb was not treated and served as a control. Vibration therapy was initiated at the end of the immobilization period, after plaster casts were removed, and continued for 2 weeks. Vibration therapy was performed for 15 min once a day. Im, immobilization; Vib, vibration.

Male Wistar strain rats (*n* = 18; age, 6 weeks) were obtained from CLEA, Japan. Rats were housed in TPX cages (two or three rats/cage), fed daily, and given free access to water. After 1 week of adaptation, rats (weight, 180–200 g) were randomly divided into four groups: (1) immobilization-only group (Im group, *n* = 3); (2) immobilization plus vibration group (Im + Vib group, *n* = 4); (3) immobilization plus no vibration treatment group (Im + non-Vib group, *n* = 4); and (4) control group (non-Im + non-Vib group, *n* = 7). Immobilization was performed for 1 week, and vibration therapy was initiated at the end of the immobilization period, after removal of plaster casts, and continued for 2 weeks. The experimental design is summarized in Figure 1.

This study was carried out in accordance with the recommendations of National Institute for Minamata Disease. The Committee on Animal Experimentation of the National Institute for Minamata Disease approved the experimental protocol.

### Immobilization

**Figure 2:**
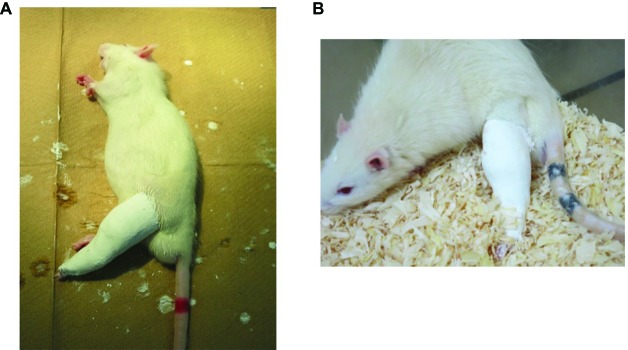
Immobilization method. **(A)** Fixation with plaster casts. Left hind limbs were fixed in the extended position, with ankle joint in full plantar flexion, using plaster casts. **(B)** An immobilized rat in a cage. One rat was housed in each cage, and animals had free access to food and water.

Immobilization was achieved as previously reported (Hamaue et al., 2015). Briefly, all rats subject to immobilization were anesthetized with isoflurane and their left hind limbs fixed in the extended position using plaster casts (Figure 2A). Ankle joints were fixed in full plantar flexion. Only left limbs were immobilized; contralateral (right) limbs were not treated and served as controls. Rats were housed in TPX cages (one rat/cage), fed daily, and given free access to water (Figure 2B). The condition of plaster casts was checked daily and changed when they were loosened or tightened.

### Plantar Vibration

**Figure 3:**
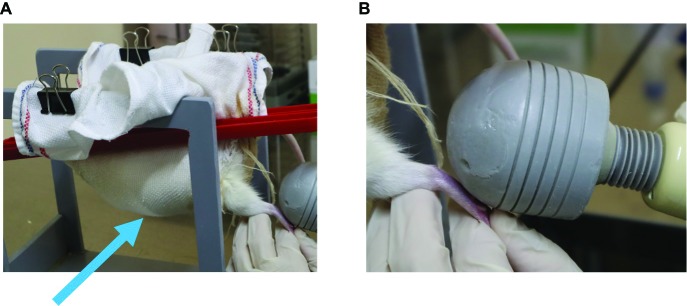
Application of vibration stimuli. **(A)** Rats were wrapped in a cloth and dangled on a wooden frame to relax their muscle tone (arrow). **(B)** The head of a hand-held vibration massager was manually applied to the sole of only the left foot.

Vibration therapy was initiated after the removal of plaster casts at the end of the immobilization period. Rats were wrapped in a cloth and dangled on a wood frame to relax their muscle tone (Figure 3A). Vibration was produced by a hand-held vibration massager (Thrive MD-01; Thrive Co., Ltd., Osaka, Japan). We selected this device in consideration of the application to clinical use because this device has shown to be clinically useful to remove plantar pain and spasticity of the lower limbs of patients with fetal-type Minamata disease (Usuki and Tohyama, 2011, 2016). The frequency that this device can produce is 90 or 110 Hz. We chose 90 Hz frequency because this frequency has been known to cause illusory movement (Goodwin et al., 1972; Naito et al., 1999). The head of a hand-held vibration massager was manually applied to only the sole of the left foot (Figure 3B). Vibratory stimuli were performed for 15 min once a day for 2 weeks. The vibration time was determined on the basis of the previous data (Usuki and Tohyama, 2011, 2016).

### Morphological Examination

At the end of the 1-week immobilization period (Im group) or after the 2-week vibration therapy period (Im + Vib and Im + non-Vib groups), rats were anesthetized with isoflurane, perfused through the heart with 0.9% ice-cold saline, and the soleus and extensor digitorum longus (EDL) muscles of both legs dissected out. We selected the soleus and EDL muscles because the rat soleus contains a high percentage of type I fibers, which has been known to be highly affected by disuse, while the EDL contains a high percentage of type IIA and IIB fibers (Pullen, 1977). A central portion of the entire muscle was immediately frozen in isopentane, cooled with liquid nitrogen, and then stored in liquid nitrogen for later histochemical examination. Serial frozen sections (8 μm) were prepared using a cryostat and stained with hematoxylin and eosin (H&E), NADH-tetrazolium reductase, and ATPase, according to the method of Dubowitz and Brooke (Dubowitz et al., 1973). Cytochrome c oxidase (CCO) was stained using the method of Seligman et al., as described previously (Usuki et al., 1998).

### Western Blotting

The peripheral parts of muscle specimens were immediately frozen in liquid nitrogen and stored at −80°C for biochemical studies. Western blotting was performed as previously described (Fujimura and Usuki, 2015, 2017). Briefly, the samples were sonicated for 5 s in tissue lysis buffer (T-PER Mammalian Protein Extraction Reagent; Pierce Biotechnology, Rockford, USA) containing Protease Inhibitor Cocktail and Phosphatase Inhibitor Cocktail 2 and 3 (Sigma-Aldrich, St Louis, USA). The samples were centrifuged (14,000 *g* for 1 h), and the supernatants were collected. The protein content was determined using the DC Protein Assay Kit II (Bio-Rad Laboratories, Hercules, USA). The cell lysates (20 μg protein) were resolved by sodium dodecyl sulfate-polyacrylamide gel electrophoresis (SDS-PAGE) on a 10% gel (Tefco, Tokyo, Japan) and transferred to nitrocellulose membranes (GE Healthcare, Buckinghamshire, UK). The membranes were then subjected to the following antibody probes: anti-MGF (Millipore, Billerica, USA); anti-IGF-I (Santa Cruz Biotechnology, CA, USA); anti-YAP1 (Novus Biologicals, Centennial, CO, USA); anti-phospho-YAP1 (Abcam, Cambridge, UK); anti-PAX7 (Cytoskeleton Incorporated, Denver, USA); anti-phospho-ERK, anti-ERK, anti-AKT, anti-phospho-AKT, anti-phospho-4EBP1, and anti-phospho-p70 S6 kinase (Cell Signaling Technologies); and anti-β-actin (Sigma-Aldrich).

### Immunohistochemistry

Frozen soleus and EDL skeletal muscle specimens were cut into 8 μm sections and placed on aminosilane-coated slides. Immunohistochemical studies were performed using anti-MGF (Millipore, Billerica, USA); anti-IGF (Santa Cruz Biotechnology, CA, USA); anti-PAX7 (R & D systems); anti-myosin 2 (MYH2; Santa Cruz Biotechnology, CA, USA); and anti-myosin 4 (MYH4; Millipore, Billerica, USA) antibodies. Biotinylated secondary antibodies were used for signal detection, using the ABC method (ABC kit; Vector, Burlingame). All immunohistochemical procedures were performed as reported previously (Higuchi et al., 1999).

### Quantitative Analysis

Myofiber cross-sectional areas were measured using the Flovel Filing System (FLOVEL Co. Ltd., Tokyo, Japan) with a MEGA PIXELS, APP-240 digital camera (Olympus). Four fields, including a total of 200 myofibers [50 myofibers per field (×200)] were measured in each rat CCO-stained skeletal muscle specimen.

PAX7-positive nuclei and MYH2-positive type IIa fibers were counted in the four fields (×200). In total, more than 300 myofibers were counted for each sample.

### Statistical Analysis

Data were analyzed by one-way ANOVA followed by Bonferroni’s multiple comparison test, or one-way Welch’s *t* test for dual comparison. Data are expressed as mean ± SEM. A difference was considered statistically significant when *p* < 0.05.

## Results

### Effects of Immobilization on Skeletal Muscle

**Figure 4:**
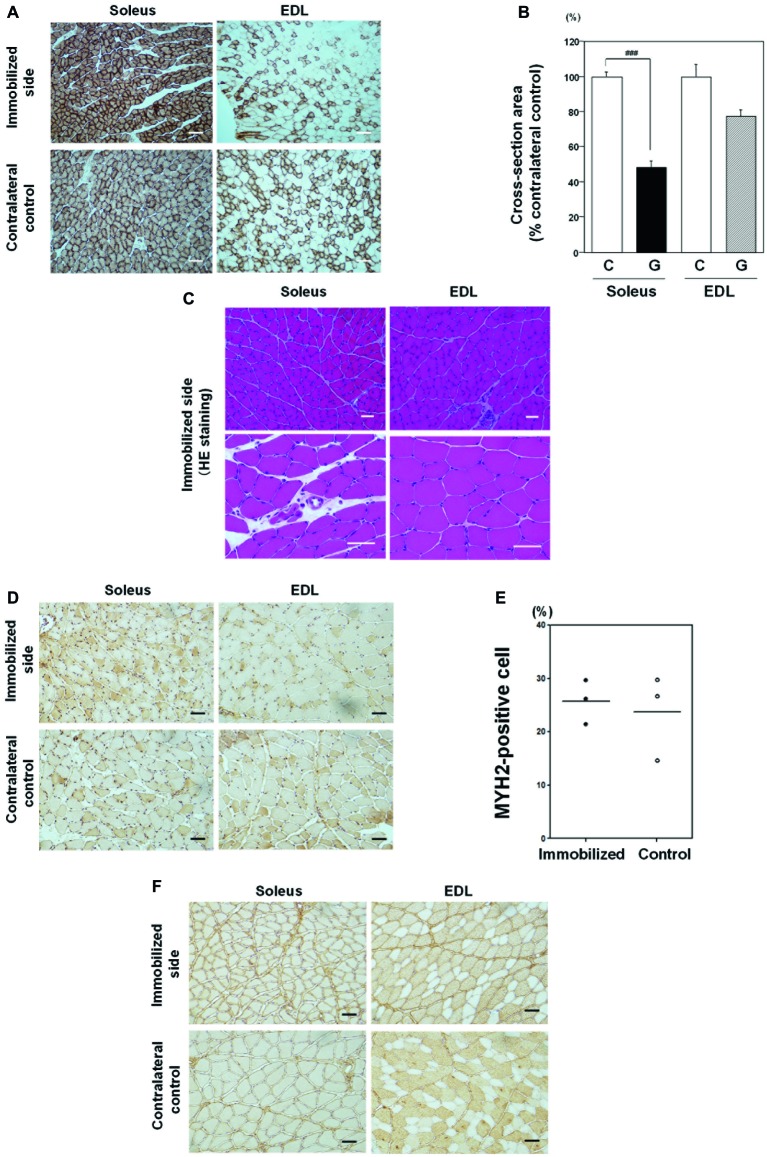
Effects of immobilization for 1 week on skeletal muscle. **(A)** Cytochrome c oxidase (CCO) activity immunostaining in soleus and EDL muscle samples from an immobilized limb (upper panel) and contralateral control samples (lower panel). Representative photographs were selected for each of the three samples. Most soleus muscle myofibers were CCO activity-rich oxidative myofibers. Immobilization for 1 week induced CCO-active oxidative myofiber atrophy in soleus skeletal muscle, compared with a contralateral non-immobilized muscle of the same rat. Bar = 100 μm. **(B)** Quantitative analysis of myofiber cross-sectional areas. Cross-sectional areas of myofibers in immobilized limb samples were significantly smaller (48.4 ± 3.3%) than those of contralateral non-immobilized soleus muscle from the same rat. Cross-sectional areas of EDL muscle from immobilized foot were not significantly different from those of contralateral non-immobilized EDL muscle from the same rat. ^###^Significantly different from the non-immobilized contralateral control (*p* < 0.001). **(C)** Hematoxylin and eosin (H&E) staining. A representative photograph was selected for each of the four samples. No necrotic fibers, increase in the number of central nuclei, or angulated fibers were observed in either soleus or EDL muscle samples. Bar = 50 μm. **(D)** Immunostaining with an anti-myosin 2 (MYH2) antibody in soleus and EDL muscles. Some MYH2-positive myofibers were detected in both immobilized and contralateral non-immobilized soleus skeletal muscle from the same rat. Bar = 50 μm. **(E)** Quantitative analysis of MYH2-positive myofibers in soleus muscle. The proportion of type IIa fiber in soleus muscle was varied among individuals. Fiber transition by immobilization for 1 week to IIa was not statistically different. **(F)** Immunostaining with an anti-myosin 4 (MYH4) antibody in soleus and EDL muscles. MYH4-positive myofibers were detected in neither immobilized nor contralateral non-immobilized soleus skeletal muscle from the same rat. Bar = 50 μm.

To investigate the effects of immobilization on slow twitch oxidative myofibers, we used CCO activity immunostaining, as CCO activity is high in mitochondria-rich oxidative myofibers. In addition, contralateral non-immobilized muscle samples from the same rats were used as controls for individual variations in muscle mass and volume because muscle mass, volume, and histology (the ratio of slow to fast muscle type, cross-sectional area) of skeletal muscle were different among individuals. As shown in Figure 4A, immobilization for 1 week induced CCO-active oxidative fiber atrophy in soleus skeletal muscle, relative to contralateral non-immobilized muscles from the same rat. By contrast, the effects of immobilization on EDL muscle were minor. Quantitative analysis of cross-sectional areas revealed that soleus muscles from immobilized limbs were significantly smaller (48.4 ± 3.3%) than those from contralateral control limbs (Figure 4B). Conversely, the cross-sectional areas of EDL muscles in immobilized limbs were not statistically significantly different to those of contralateral non-immobilized EDL muscles from the same rat. No myopathic changes, including necrotic fibers, inflammatory change, or increase in the number of central nuclei, were detected in either the soleus or EDL skeletal muscles after immobilization for 1 week (Figure 4C). Non-grouping scattered atrophic angulated fibers were also detected in both types of skeletal muscles. These findings indicate that immobilization with a cast for 1 week induced rat oxidative myofiber-specific atrophy, without myopathic changes.

Next, we examined the appearance of type II fibers in soleus muscle to investigate the effects of immobilization on fiber transition (Figures 4D,F). Quantitative analysis of non-immobilized control soleus muscle showed that type IIa myofibers, stained with anti-MYH2 antibody, comprised 15–29% (Figure 4E), while type IIb, stained with anti-MYH4 antibody, was absent (0%; Figure 4F), consistent with previous reports (Pullen, 1977). The proportion of type IIa fiber in soleus muscle was varied among individuals. Fiber transition by immobilization for 1 week to type IIb was not observed, and transition to type IIa was not statistically different (Figure 4E).

### Effects of Vibration Therapy on Immobilization-Induced Muscle Atrophy

To investigate the therapeutic effect of mechanical stimulation, vibration stimulus to the plantar fascia of an atrophic limb was performed for 2 weeks using a hand-held vibration massager, after the removal of plaster casts at the end of immobilization period.

**Figure 5:**
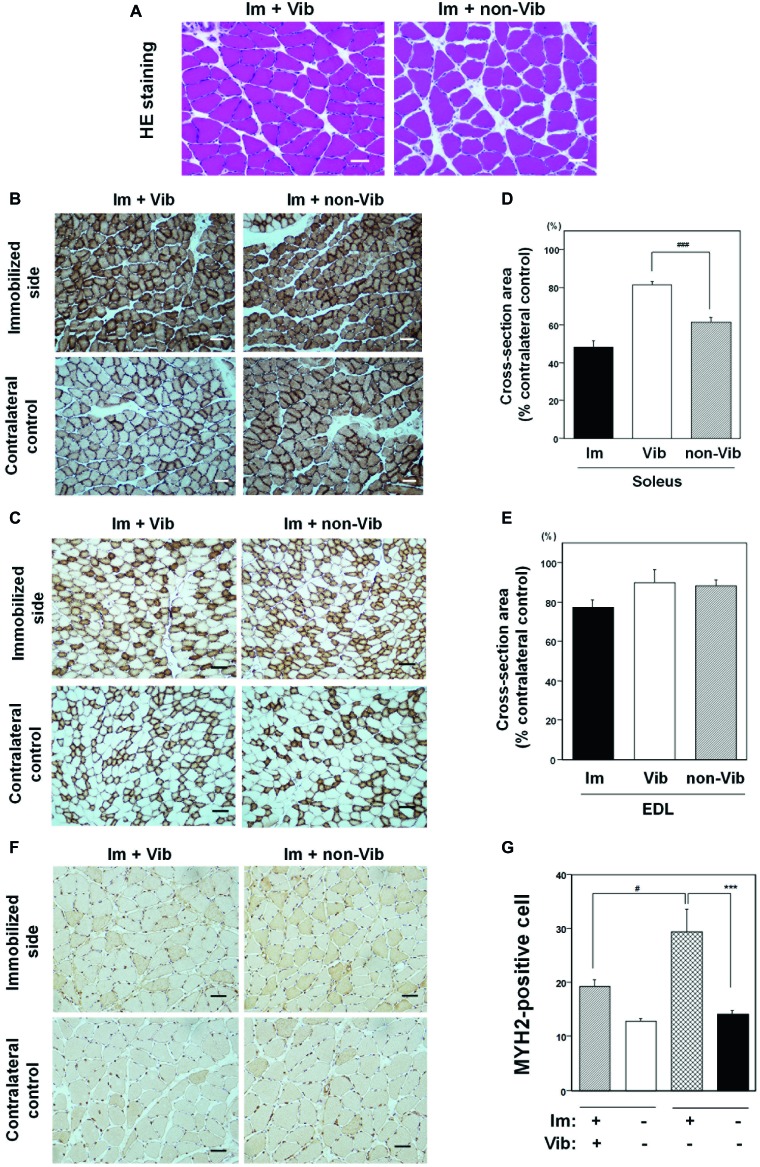
Effects of vibration therapy for 2 weeks on immobilization-induced oxidative myofiber atrophy. **(A)** Hematoxylin and eosin (H&E) staining. An increase in the interstitial area was observed in the non-vibration group (right panel). Representative photographs were selected for each of the four samples. Bar = 50 μm. **(B,C)** Immunostaining for CCO activity in soleus **(B)** and EDL **(C)** muscles. Representative photographs of vibration-treated (left panels), and vibration-untreated (right panels), immobilized limbs (upper panels), and contralateral controls (lower panels), were selected for each of the four samples. Vibration therapy caused a recovery effect on soleus skeletal muscle atrophy. Bar = 50 μm. **(D,E)** Quantitative analysis of the cross-sectional areas of myofibers in soleus **(D)** and EDL **(E)** muscle. The cross-sectional areas (% contralateral control) of vibration-treated immobilized limb samples were significantly larger (81.5 ± 1.7%) than those in vibration-untreated immobilized limbs (61.7 ± 2.3%), in soleus muscle. Cross-sectional areas (% contralateral control) of vibration-treated immobilized limb samples in EDL muscle were not significantly different from those in vibration-untreated EDL muscle. The cross-sectional areas of soleus or EDL muscle after a 1-week immobilization were also shown in each figure. ^###^Significantly different from vibration-untreated immobilized muscle by one-way Welch’s *t* test (*p* < 0.005). **(F)** Immunostaining with an anti-myosin 2 (MYH2) antibody in soleus muscle. An increase in MYH2-positive myofibers was detected in immobilized muscle compared to contralateral non-immobilized soleus skeletal muscle from the same rat. Bar = 50 μm. **(G)** Quantitative analysis of MYH2-positive myofibers in soleus muscle. Fiber transition to IIa was statistically different in Im + non-Vib group but not in Im + Vib group. The fiber transition to type IIa in soleus muscle was statistically suppressed in Im + Vib group compared to that in Im + non-Vib group. Data are represented as the mean ± SEM (*n* = 4). ^***^Significantly different from non-immobilized contralateral control muscle by a one-way ANOVA followed by Bonferroni’s multiple comparison test (*p* < 0.001). ^#^Significantly different from vibration-untreated immobilized rats by a one-way ANOVA followed by Bonferroni’s multiple comparison test (*p* < 0.05).

The mean ± SE body weight of the Im + Vib group (369.9 ± 14.5 g; *n* = 4) at the end of 2 weeks of vibration therapy did not differ significantly from that of the Im + non-Vib group (381.8 ± 19.9 g; *n* = 4). Vibration therapy caused a remarkable effect on soleus skeletal muscle atrophy. Morphological examination revealed an increase in the interstitial area in soleus muscle in Im + non-Vib group, while no similar change was observed in (Im + Vib) group (Figure 5A). In addition, vibration therapy caused fast recovery from oxidative myofiber atrophy (Figure 5B). Quantitative analysis of myofibers stained for CCO activity demonstrated that plantar vibration caused a significant increase in the cross-sectional area of the soleus skeletal muscle in the (Im + Vib) group compared with that in (Im + non-Vib) group (Figure 5D). The findings indicate that the recovery from immobilization-induced soleus skeletal muscle atrophy was facilitated in Im + Vib group than that in Im + non-Vib (Sham group). By contrast, the cross-sectional area of the EDL skeletal muscle did not differ significantly between the two groups, suggesting that recovery from type II fiber atrophy may be rapid even where there is some immobilization-induced type II fiber atrophy (Figures 5C,E).

Study on fiber transition from slow to fast type in soleus muscle was performed using anti-MYH2 antibody. Quantitative analysis revealed that MYH2-positive myofibers were significantly increased in immobilized soleus muscle from that in contralateral control muscle in Im + non-Vib group but not significantly different in Im + Vib group. The fiber transition to type IIa in soleus muscle was statistically suppressed in Im + Vib group compared to that in Im + non-Vib group (Figures 5F,G).

### Western Blot Analyses of Factors Related to Muscle Growth and Atrophy

**Figure 6:**
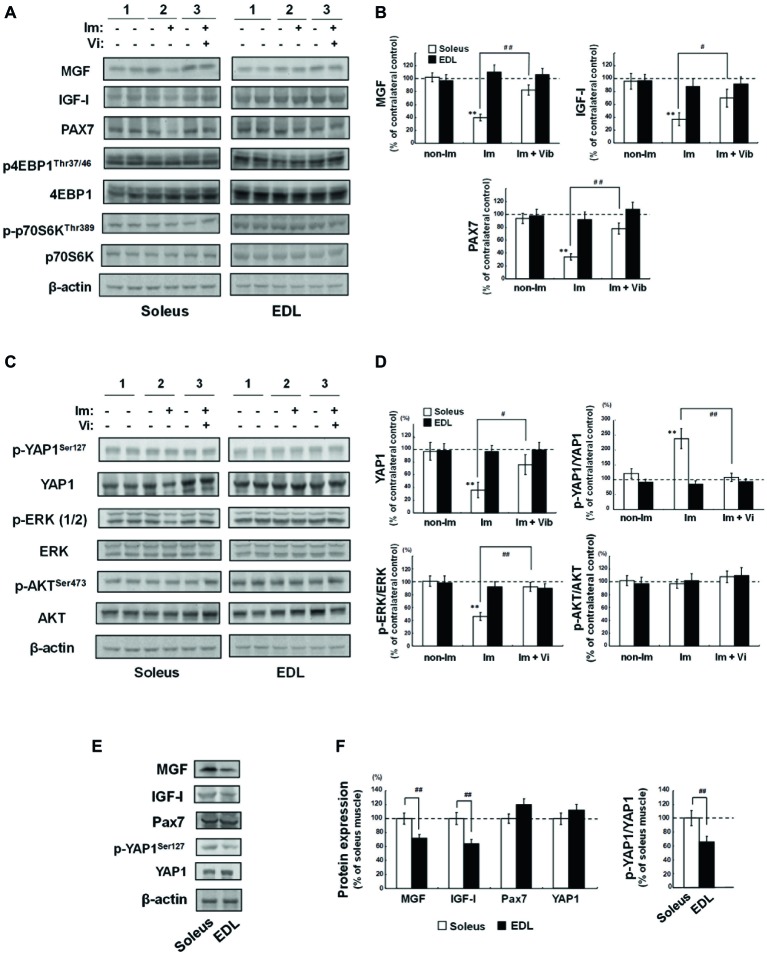
Western blot analyses of soleus and EDL muscle extracts. 1) Non-Im, non-immobilized vibration-untreated control rat; 2) Im, immobilized vibration-untreated rat; 3) Im + Vib, immobilized vibration-treated rat. **(A)** Western blot analyses of factors related to muscle growth and atrophy. Images are representative of four independent experiments. Prepared muscle samples were analyzed using the indicated antibody probes. Cropped blots are shown; all gels were run under the same experimental conditions. **(B)** Densitometric quantification of the bands shown in **(A)**. The histogram depicts the levels of MGF, IGF-I, or PAX7 normalized to those of β-actin, represented as the percentage increase over non-immobilized contralateral control samples from the same rat. Data are represented as the mean ± SEM (*n* = 4). ^**^Significantly different from non-immobilized contralateral control muscle by a one-way ANOVA followed by Bonferroni’s multiple comparison test (*p* < 0.01). ^#, ##^Significantly different from vibration-untreated immobilized rats by a one-way ANOVA followed by Bonferroni’s multiple comparison test (^#^*p* < 0.05, ^##^*p* < 0.01). **(C)** Western blot analyses of YAP1, ERK, and AKT. Photographs are representative of four independent experiments. Prepared muscle samples were analyzed using the indicated antibody probes. Cropped blots are shown; all gels were run under the same experimental conditions. **(D)** Densitometric quantification of the bands shown in **(C)**. The histogram depicts the levels of YAP1 normalized to those of β-actin and p-YAP1, p-ERK, or p-AKT levels normalized to those of YAP1, ERK, or AKT, respectively, represented as the percentage increase over non-immobilized contralateral control samples from the same rat. Values are the means ± SEM (*n* = 4). ^**^Significantly different from non-immobilized contralateral control muscle by a one-way ANOVA followed by Bonferroni’s multiple comparison test (*p* < 0.01). ^#, ##^Significantly different from vibration-untreated immobilized rats by a one-way ANOVA followed by Bonferroni’s multiple comparison test (^#^*p* < 0.05, ^##^*p* < 0.01). **(E,F)** Comparison of the basic level of MGF, IGF-I, PAX7, and YAP1, between soleus and EDL muscles. **(E)** Western blot analyses of MGF, IGF-I, PAX7, p-YAP1, and YAP1. Photographs are representative of four independent experiments. Prepared muscle samples were analyzed using the indicated antibody probes. Cropped blots are shown; all gels were run under the same experimental conditions. **(F)** Densitometric quantification of the bands shown in **(E)**. The histogram depicts the level of the indicated protein expression normalized to β-actin (MGF, IGF-I, and PAX7) or YAP1 (p-YAP1) represented as the percentage increase over soleus muscle from the same rat. Values are the means ± SEM (*n* = 4). ^##^Significantly different from EDL muscle by one-way Welch’s *t* test (*p* < 0.01).

To investigate the mechanism underlying the therapeutic effects of vibration stimuli on immobilization-induced soleus skeletal muscle atrophy, we first focused on the MGF, systemic variant of IGF-I, and muscle stem cells (Goldspink, 1999; Kandalla et al., 2011; Brooks and Myburgh, 2014; Gnimassou et al., 2017; Fukada, 2018). MGF has been known to be a mechanical stress-induced factor (Yang et al., 1996; Goldspink, 2005), and muscle also expresses the systemic type of IGF-I (Goldspink, 2005). Western blot analysis revealed a decrease in levels of MGF and IGF-1 in (Im + non-Vib) soleus muscle [lane 4, left panel vs. lane 3, left panel (contralateral control); Figure 6A]. Vibration stimuli restored the decreased expression of MGF and IGF-1 (lane 6, left panel; Figure 6A). Furthermore, vibration stimuli restored immobilization-induced downregulation of Pax7, which is expressed in satellite cells (Seale et al., 2000), in soleus skeletal muscle (lanes 4 and 6, left panel; Figure 6A); similar changes were not detected in EDL muscle. Quantitative analyses revealed the results (Figure 6B). Although we examined the expression of phospho-4EBP1 and phospho-p70 S6 kinase, both of which are factors involved in the mTOR pathway, we did not observe any change in the expression of these factors.

Next, we examined the mechanotransduction protein, YAP1 (Dupont et al., 2011), the mitogen-activated protein kinase (MAPK), and AKT pathways, which transduce extracellular signals to provoke intracellular responses. As shown in Figure 6C, a decrease in YAP1 level was observed in (Im + non-Vib) soleus muscle [lane 4, left panel vs. lane 3, left panel (contralateral control)]. However, no change was observed in the level of YAP1 phosphorylation (YAP1^Ser127^). As shown in Figure 6D, p-YAP1/YAP1 ratio was increased in (Im + non-Vib) soleus muscle, whereas no change in p-YAP1/YAP1 ratio was observed in (Im + Vib) soleus muscle because of an increase in YAP1. Since p-YAP1 has been known to be an inactive form of YAP1 (Yu and Guan, 2013), the results indicated that vibration stimuli restored immobilization-induced inactivation of YAP1 pathway. In addition, phosphorylation of ERK1/2, which regulates cell growth and cell cycle progression, was suppressed in (Im + non-Vib) soleus muscle, whereas it was restored after vibration stimuli. By contrast, phosphorylation of AKT did not alter in response to vibration therapy. Moreover, these changes were not observed in EDL muscle.

The basic level of MGF, YAP1, IGF-I, or PAX7 was compared between soleus and EDL muscles (Figures 6E,F). Quantification analysis indicated that the levels of MGF and IGF-I, and p-YAP1/YAP1 ratio were significantly higher in soleus muscle than those in EDL muscle, whereas the level of PAX7 was not significantly different between them.

### Immunohistochemical Analyses of Factors Related to Muscle Growth and Atrophy

**Figure 7:**
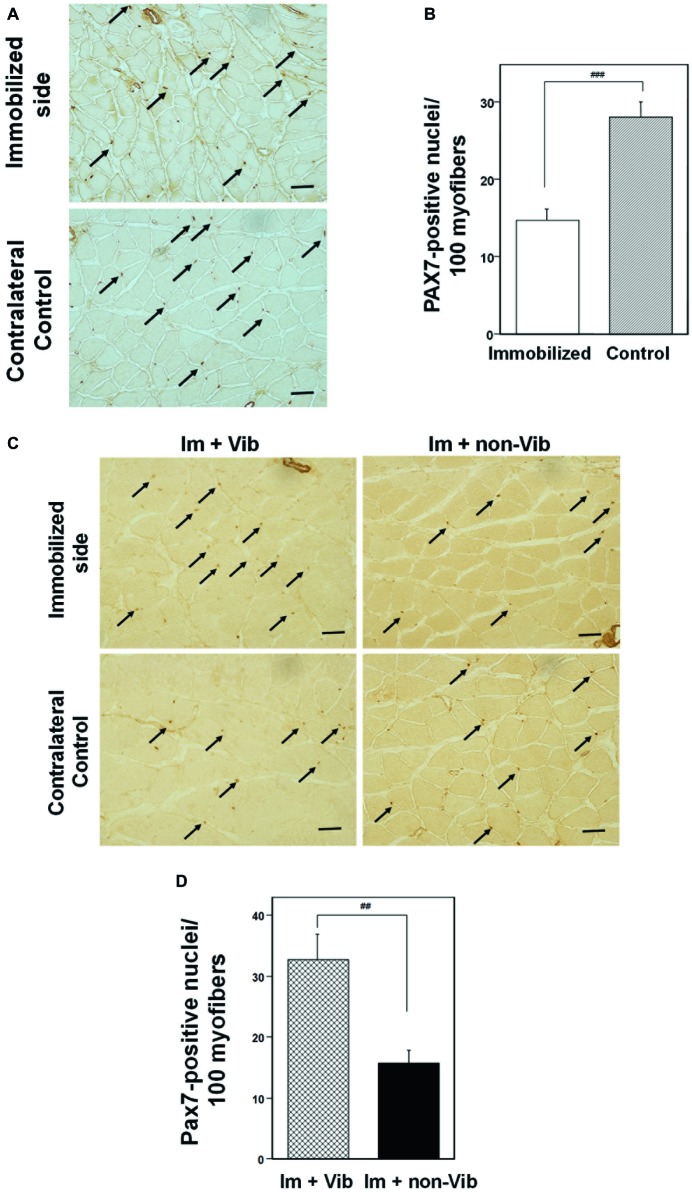
Immunohistochemistry analysis of soleus skeletal muscle with an anti-PAX7. **(A)** Effects of immobilization for 1 week on the PAX7 expression in satellite cells. PAX7-positive satellite cells are indicated by arrows. Representative photographs were selected for each of the three samples. Bar = 50 μm. **(B)** Quantitative analysis of cross-sectional areas. PAX7-positive nuclei were counted in four fields (×200). The histogram depicts PAX7-positive nuclei per 100 myofibers. Numbers of PAX7-positive satellite cells were significantly lower in samples from limbs immobilized for 1 week than those in samples from contralateral non-immobilized soleus muscle. ^###^Significantly different from non-immobilized contralateral control by one-way Welch’s *t* test (*p* < 0.001). **(C)** Immunostaining of an immobilized limb sample (upper panel) and a contralateral control (lower panel) with an anti-PAX7 antibody. Vibration stimuli induced PAX7-positive satellite cells (arrow) in the immobilized muscle. Representative photographs were selected for each of the four samples. Im + Vib, vibration-treated immobilized muscle; Im + non-Vib, vibration-untreated immobilized muscle. Bar = 50 μm. **(D)** Quantitative analysis of cross-sectional areas in soleus muscle. PAX7-positive nuclei were counted in four fields (×200). The histogram depicts PAX7-positive nuclei per 100 myofibers. Numbers of PAX7-positive satellite cells were significantly higher in soleus muscle in Im + Vib group than those in samples from Im + non-Vib group. ^##^Significantly different from vibration-untreated muscle by one-way Welch’s *t* test (*p* < 0.01).

The effect of immobilization for 1 week on satellite cells was investigated by immunohistochemical analysis of PAX7 (Figure 7A). Quantitative analysis of PAX7 levels revealed that immobilization induced a significant decrease in the number of PAX7-positive satellite cells compared with non-immobilized soleus muscle from the same rat (Figure 7B). Investigation of the effect of vibration on PAX7 expression of immunohistochemistry generated similar results to those of western blot analyses (Figures 7C,D). Quantitative analysis of PAX7 levels per myofibers revealed that vibration stimuli significantly induced PAX7-positive satellite cells in atrophic soleus muscle compared to non-vibration muscle (Figure 7D).

## Discussion

In this study, we demonstrate for the first time that local vibration stimulation of the plantar fascia can facilitate recovery from immobilization-induced soleus skeletal muscle atrophy in rats and suppress the fiber transition from slow oxidative to fast glycolytic fiber types (Figures 5A–G). Immunohistochemistry and western blotting analyses demonstrated that vibration stimulation of the plantar fascia upregulates MGF, systemic IGF-Ia, and YAP1 expression, without alteration of phosphorylated-YAP1^Serine127^ levels (Figure 6). Immobilization with a plaster cast for 1 week caused a decrease in the number of PAX7-positive satellite cells in soleus skeletal muscle (Figures 7A,B), while vibration stimulation upregulated the number of PAX7-positive satellite cells in atrophic soleus muscle induced by immobilization (Figures 6A–D). These findings indicate that the mechanisms underlying the therapeutic effects of vibration stimuli on disuse atrophy involve the induction of MGF, systemic IGF-Ia, YAP1, and PAX7.

MGF, a splice variant of the IGF-I gene, is a regulator of local muscle growth following the physical activity (Yang et al., 1996). Muscle can express two splice variants of the IGF-I gene, MGF, and systemic variant of IGF-I (IGF-IEa; Goldspink, 1999). MGF and IGF-IEa are both shown positive regulators of muscle hypertrophy (Goldspink, 1999). MGF is an autocrine growth factor, which is regulated by mechanical stimuli and its expression precedes that of IGF-IEa to activate muscle satellite cell proliferation (Hill et al., 2003). IGF-IEa was postulated to have a delayed effect that is sustained during the later phase of regeneration (Hill et al., 2003); however, a recent *in vitro* study demonstrated that MGF alone has a marked ability to enhance satellite cell activation, proliferation, and fusion for muscle repair (Kandalla et al., 2011). Although MGF and IGF-IEa both contain exons 3 and 4 of the *IGF-I* gene, which encode the IGF-I receptor ligand domain, induction of satellite cell proliferation by MGF does not proceed *via* an IGF-I receptor (Yang and Goldspink, 2002). MGF activates phosphorylation of ERK 1/2, whereas IGF-IEa can phosphorylate both ERK 1/2 and AKT (Philippou et al., 2009). In this study, vibration therapy enhanced phosphorylation of ERK 1/2 but not AKT (Figure 6C), indicating that MGF has an important role in inducing generation of PAX7-positive satellite cells and recovery from immobilization-induced soleus skeletal muscle atrophy.

YAP1 is a transcriptional co-factor that is negatively regulated *via* phosphorylation at Ser127. Mammalian YAP was first characterized in 1995 and is ubiquitously expressed, including in skeletal muscle (Sudol et al., 1995). YAP1 has been identified as a sensor and a mediator of mechanical cues from the cellular microenvironment (Dupont et al., 2011). In its active state, YAP1 localizes to the nucleus and regulates the activity of several transcription factors (Fischer et al., 2016). In quiescent satellite cells, YAP1 levels are low, whereas it is robustly expressed in activated satellite cells (Judson et al., 2012). YAP1 expression increases during satellite cell activation and remains at high levels, until after decision for the cells to differentiate or self-renewal is made (Judson et al., 2012). Furthermore, YAP is a critical regulator of skeletal muscle fiber size (Watt et al., 2015). In this study, we demonstrate for the first time that vibration stimuli to the plantar fascia can restore immobilization-induced inactivation of YAP1 pathway followed by an increase in YAP1 expression. Although no change in the level of phosphorylated YAP1^Ser127^ was observed in immobilization, p-YAP1/YAP1 ratio was increased in (Im + non-Vib) soleus muscle. Vibration-induced increase in total YAP1 resulted in no change in p-YAP1/YAP1 ratio in (Im + Vib) soleus muscle. Since p-YAP1 has been known to be an inactive form of YAP1 (Yu and Guan, 2013), the results indicate that vibration stimuli are effective to restore immobilization-induced inactivation of YAP1 pathway. A previous report indicated that relatively short-term activation of YAP1, perhaps in a pulsatile manner, is sufficient to induce muscle hypertrophy (Goodman et al., 2015). Our findings suggest that cyclic vibration stimuli (90 Hz, 15 min, once a day) are useful to induce the expression of YAP1, leading to the recovery of muscular atrophy.

Collectively, our results indicate that cyclic vibration stimuli can effectively activate satellite cells and facilitate recovery from immobilization-induced skeletal muscle atrophy through the upregulation of MGF and YAP1 expression. To date, vibration therapy has been shown to have preventive effects against immobilization-induced hypersensitivity and decreased bone mineral density and therapeutic effects on plantar pain and spasticity of the lower limbs (Hamaue et al., 2015; Usuki and Tohyama, 2016; Huang et al., 2018). In this study, we further demonstrate the therapeutic effects of vibration therapy on immobilization-induced oxidative myofiber atrophy. Our data show that vibration-induced upregulation of MGF and YAP1 contributes to the activation of PAX7-positive satellite cells, leading to recovery from oxidative myofiber atrophy.

The device we used in this study was a commercial hand-held vibration massager that could generate an effective frequency of 90 Hz. Since this method is inexpensive, non-invasive, and convenient, we believe that it could be widely used to facilitate recovery from disuse-induced skeletal muscle atrophy.

## Data Availability

No datasets were generated or analyzed for this study.

## Ethics Statement

This study was not exempted from the Committee on Animal Experimentation of the National Institute for Minamata Disease. This study was carried out in accordance with the recommendations of National Institute for Minamata Disease. The Committee on Animal Experimentation of the National Institute for Minamata Disease approved the experimental protocol.

## Author Contributions

MF, AN, and FU conceived and designed the experiments. MF and AN performed the experiments. FU, MF, and IH analyzed the data. AN, MF, JN, MO, and IH contributed materials and analysis tools. FU and MF wrote the manuscript. All authors reviewed the manuscript.

### Conflict of Interest Statement

The authors declare that the research was conducted in the absence of any commercial or financial relationships that could be construed as a potential conflict of interest.
